# Effect of a community health worker delivered health, nutrition and responsive stimulation package and conditional cash transfers on child development and growth in rural Tanzania: protocol for a cluster-randomized trial

**DOI:** 10.1186/s12889-019-7008-6

**Published:** 2019-05-27

**Authors:** Christopher R. Sudfeld, Lilia Bliznashka, Geofrey Ashery, Aisha K. Yousafzai, Honorati Masanja

**Affiliations:** 1 Department of Global Health and Population, Harvard T.H. Chan School of Public Health, Boston, MA USA; 2 Department of Nutrition, Harvard T.H. Chan School of Public Health, Boston, MA USA; 3 Ifakara Health Institute, Dar es Salaam, Tanzania

**Keywords:** Nutrition, Child development, Community health worker, Cash transfer

## Abstract

**Background:**

Child health, nutrition, and responsive stimulation interventions have been developed to improve child survival, growth and development outcomes in low- and middle-income countries. Nevertheless, research on integrated implementation approaches to deliver and promote uptake of these interventions is needed, particularly in sub-Saharan Africa.

**Methods/design:**

We will conduct a cluster-randomized controlled trial of a supply-side community health worker (CHW) delivered child health, nutrition, and responsive stimulation intervention alone and in combination with a demand-side conditional cash transfer (CCT) intervention to promote antenatal care and child growth monitoring attendance in rural Morogoro region, Tanzania. Twelve village clusters will be randomly assigned to one of the three trial arms: (1) CHW, (2) CHW + CCT, or (3) Control. Mothers (or another primary caregiver) residing in study villages are eligible for trial enrollment if they are currently pregnant or have a child < 1 year of age at the time of enrollment. For the duration of the trial, CHWs will visit households once every 4–6 weeks to deliver the intervention package and CCTs will be provided for documented antenatal care and routine child health and growth monitoring clinic visits. Participants will be followed-up at 9 months (midline) and 18 months (endline) post-randomization. The primary outcomes of the trial are child development assessed by the Bayley Scales of Infant and Toddler Development (BSID-III) and child height-for-age z-score. Secondary outcomes include a range of maternal, child and household outcomes.

**Discussion:**

This trial will provide evidence on the effect of CHWs and conditional cash transfers on child growth and development. The results of the trial may be generalizable to similar settings in sub-Saharan Africa.

**Trial registration:**

ISRCTN10323949, Retrospectively registered on October 3, 2017.

## Background

In 2010, it was estimated that over 300 million children under the age of five were stunted and approximately 250 million children in low- and middle-income countries (LMIC) did not reach their development potential [1, 2]. Suboptimal development during infancy may persist through childhood and lead to poor schooling achievement and human capital outcomes later in life [3, 4]. As a result, alleviating early life adversity and supporting child development may help reach long-term health and poverty reduction goals in LMIC.

Intervention packages that include multiple health, nutrition, and responsive stimulation components may be impactful in promoting children’s growth and development [5]. Further, community-based and integrated strategies may provide greater intervention coverage, impact, and address the limited human and financial resources in LMIC [6]. Community health worker (CHWs) can increase the rate of facility births, uptake of child immunizations, improve breastfeeding practices and reduce morbidity and mortality for newborns; however, the effect of CHW-delivered interventions on child growth outcomes remains unclear [7–9]. Community-based approaches to deliver responsive stimulation interventions have been shown to improve child development outcomes in LMICs [10]. Nevertheless, evidence on the effect of CHW delivered child health, nutrition, and responsive stimulation intervention packages on child growth and development outcomes in sub-Saharan Africa remains sparse.

Conditional cash transfers (CCTs) have shown promise in promoting behavioral changes related to health and education outcomes in LMICs [11]. CCTs tied to health service utilization have generally shown positive effects on antenatal care and well-child clinic attendance; however, evidence on child health, nutrition and development outcomes is mixed and limited in the context of sub-Saharan Africa [11, 12]. A meta-analysis of 17 CCTs programs, with the majority of trials conducted in South America, found no effect on child growth outcomes [13]. Nevertheless, studies in Columbia and Mexico have indicated that integration of CCTs with responsive stimulation and group-based parenting-support interventions can improve child development outcomes [14, 15]. Therefore, the effect of CCTs alone and in combination with integrated child health, nutrition, and stimulation interventions remains unclear.

We present the protocol for a cluster-randomized controlled trial of a supply-side CHW delivered health, nutrition, and responsive stimulation intervention alone and in combination with a demand-side CCT intervention to promote antenatal care and routine child growth monitoring visit attendance in rural Tanzania. The co-primary outcomes of the trial are child development and linear growth. We will also examine a range of secondary maternal, child, and household outcomes. This trial is intended to inform whether policymakers in Tanzania and similar settings should consider adding a responsive stimulation to CHW curriculums and whether coordination of CHW and CCT programs can improve child growth and development.

## Methods / design

We will conduct a proof-of-concept cluster RCT to examine the effect of a supply-side CHW delivered health, nutrition, and responsive stimulation intervention alone and in combination with a demand-side CCT tied to antenatal care and routine child growth monitoring clinic attendance (trial registration: ISRCTN10323949). The trial protocol was developed by collaborators at the Ifakara Health Institute in Tanzania and the Harvard T.H. Chan School of Public Health in the United States. This protocol (version 2.0/17 March 2017) was written in concordance with the Standard Protocol Items: Recommendations for Interventional Trials [16].

**Fig. 1:**
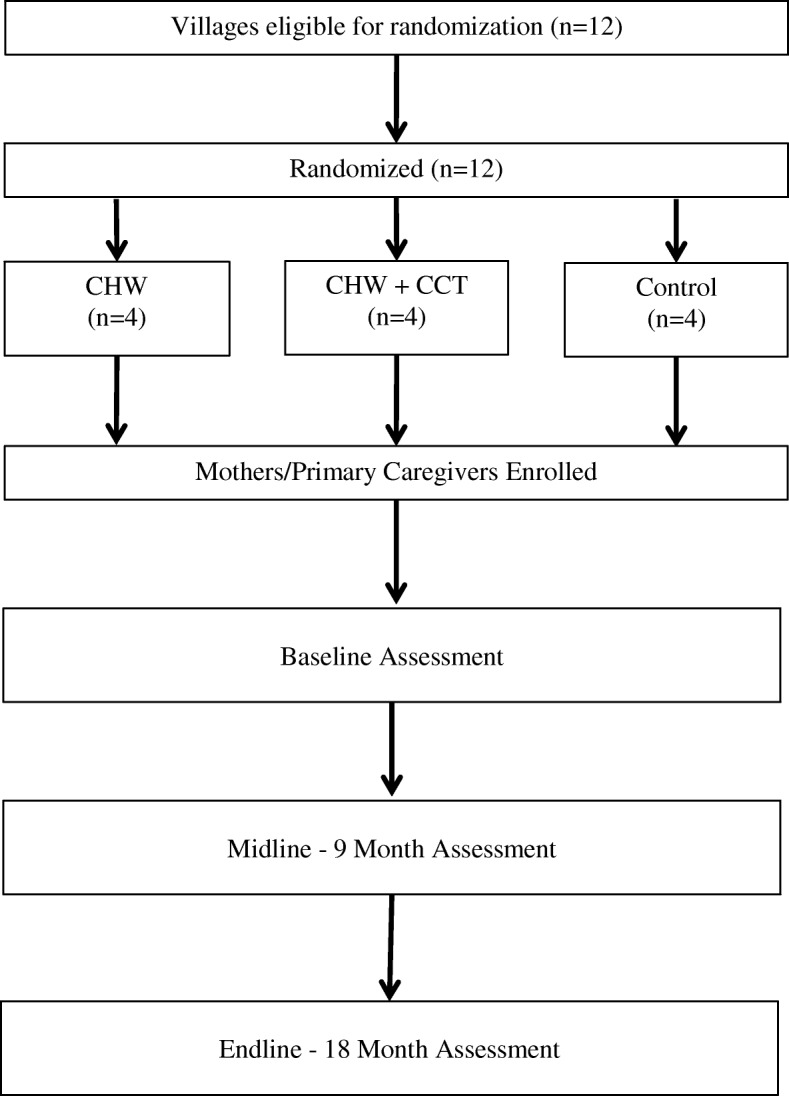
Trial flow diagram

The trial flow diagram is presented in Fig. 1. Twelve village clusters in the Ifakara Health and Demographic Surveillance (HDSS) system were randomly assigned to one of the three trial arms: (i) CHW, (ii) CHW + CCT, or (iii) Control. The CHW arm will receive the CHW delivered health, nutrition and responsive stimulation intervention for the duration of the trial. The CHW + CCT arm will receive the same CHW intervention combined with a CCT intervention for the duration of the trial. The control arm will have access to the existing clinic-based health, nutrition and development services; no quality improvement to existing serves will be provided. A total of ~ 600 mother/caregiver-child dyads residing in the study villages will be enrolled. Study participants will be assessed at midline (9 months of follow-up) and endline (18 months of follow-up) for primary and secondary outcomes.

### Primary and secondary outcomes

The co-primary outcomes of the trial are (1) child development composite scores and (2) child linear growth as assessed by height/length-for-age z-score (HAZ) at trial endline. We will also assess effect of the interventions on child development and child linear growth at midline as secondary outcomes. Additional secondary outcomes include a range of household, maternal, and child outcomes that may be affected by either the CHW and/or CCTs interventions. Secondary outcomes assessed at midline and endline include: (i) child weight-for-age z-score (WAZ), (ii) child weight-for-height/length z-score (WHZ), (iii) child head circumference-for-age z-score (HCAZ), (iv) child mid-upper arm circumference (MUAC) z-score, (v) antenatal and growth monitoring clinic visit attendance, (vi) child morbidities, (vii) child hospitalization, (viii) maternal depression and anxiety, (ix) maternal social support, (x) women’s empowerment, (xi) caregiver knowledge of child development, (xii) caregiver report on engagement in stimulation activities and (xiii) household food security.

### Trial setting

This trial is being conducted in the Ifakara Health Institute HDSS in the Kilombero and Ulanga districts in the Morogoro region of Tanzania [17]. The site is located between latitudes 8°00′S and 8°35′S and longitude 35°58′E to 36°48′E. The HDSS study area is located approximately 450 km by road from Dar es Salaam with a catchment population of ~ 400,000 people. The study area is predominately rural, and the majority of residents are subsistence farmers. A recent study conducted in the Ifakara HDSS found a 16.9% prevalence of low birthweight (< 2500 g) and a 36.2% stunting prevalence (HAZ < -2) among children 18–36 months of age [18].

### Trial population

Women/caregivers and their infants (if applicable) will be eligible for trial enrollment if they met the following inclusion criteria at the time of the enrollment visit: (1) permanent resident of study village, (2) currently pregnant (self-reported) or have a child < 1 year of age, (3) and provide written consent for enrollment. Women/caregivers will be excluded from participation if they: (1) are currently enrolled in any other clinical trial or intervention study, or (2) do not provide informed consent. Written informed consent will be obtained for all participants.

### Sample size

Sample size calculations were based on randomization of 12 clusters with a cluster size of approximately 50 mother/caregiver-child pairs. We assumed a nominal Type I error rate (alpha) of 0.05 and assume a coefficient of variation of 0.49 an intra-cluster correlations of 0.01. We will have 80% power to detect a standardized mean difference (SMD) of 0.40 standard deviations in normalized anthropometric outcomes (HAZ, WHZ, WAZ), and 0.53 standard deviations of development z-scores. These are quite large effect sizes; however, this is a proof-of-concept trial and there is potential of the intervention package and the combination of CHWs and CCT to provide a sizable impact on child growth and development.

### Randomization and interventions

Village clusters will be allocated in a 1:1:1 ratio to one of the three trials arms: (i) CHW, (ii) CHW + CCT, or (iii) Control. Stratified randomization will be done by a non-study statistician in Boston, USA according to a computer-generated randomization list with sequence blocks of three. Randomization will be stratified by semi-urban (six villages) and rural (six villages) villages to increase the likelihood of baseline balance.

#### CHW delivered child health, nutrition and responsive stimulation intervention

The CHW and CHW + CCT arms will receive the same supply-side CHW intervention. The project will recruit female CHWs living in the study villages catchment area who had completed a one-year government certified CHW program prior to being selected for the intervention. The government CHW curriculum included two semesters that each covered seven topics. The first semesters covered: (1) fundamentals of communication and customer service, (2) infection prevention and control, (3) management of health care facility, (4) computer application, (5) citizenship and gender, (6) management information systems, and (7) basic life support skills. The second semester covered: (1) fundamentals of social work, (2) disease prevention and control, (3) community-based reproductive, maternal and child health services, (4) community-based health promotion, (5) home-based care, (6) basics of entrepreneurship and life skills, and (7) health facility and community disease management. The main infant intervention components of the CHW curriculum include: 1) community-case management of under-5 childhood illness per Integrated Management of Childhood Illness (IMCI); 2) antenatal and postnatal pregnancy, delivery and essential newborn care counseling and danger signs identification; 3) family planning; 4) and emergency and routine referrals to facilities. The CHWs will be assigned to the study villages at the start of the trial. The salary for the CHWs is ~ 600,000 Tanzanian shillings (~$230) per month.

We will train the CHWs to deliver a responsive stimulation intervention that was integrated into the routine CHW home visit program. The responsive stimulation intervention is a Tanzanian and Swahili adapted version of the UNICEF and WHO Care for Development package [19]. The intervention includes communication of essential early childhood development knowledge, promotion of caregivers’ sensitivity and responsiveness, developmentally appropriate play and communication activities, toy making, and problem solving. Caregivers are provided the opportunity to try out responsive stimulation activities with their young child and receive feedback and coaching from the CHW. The CHWs will received a one-week training in September 2017 on the responsive stimulation intervention that included integrated classroom and practical sessions.

CHWs will conduct home visits every 4–6 weeks throughout the trial follow-up period (18 months) to deliver the health, nutrition and responsive stimulation intervention package. The intended duration of the visit is 30–60 min per household, dependent on the health and well-being of the mother and child. One study coordinator will supervise the CHWs throughout the intervention. CHWs will met individually with the study coordinator biweekly to report visit completion. The study coordinator will accompany CHWs at least once per month to supervise home visits and provide feedback on intervention delivery. CHWs will also keep administrative records including the duration of home visits and the composition of messages delivered at each visit that will be reviewed by the study coordinator once a month. Monthly meetings will be held with all CHWs and the study coordinator to discuss progress and troubleshoot emerging challenges. A three-day refresher training on the responsive stimulation intervention will be conducted at trial midline (9 months). We will conduct in-depth qualitative interviews with each CHW at intervention completion to document their experiences and suggestions for improvement.

#### Conditional cash transfer intervention

CHWs will also provide a demand-side CCT intervention to mothers/caregivers in the CHW + CCT arm. The conditions for the cash transfers are documented attendance of antenatal care or routine well-child health and growth monitoring clinic visits. Tanzania currently uses a focused antenatal care model with four visits; one before 16 weeks, at 20–24 weeks, at 28–32 weeks, and at 36 weeks gestation; postnatal child health and growth monitoring clinic visits are recommended to occur monthly until 2 years of age and then quarterly until 5 years [20, 21]. Reproductive and child health cards will be checked at CHW visits to document clinic visit attendance. CHWs will provide the CCTs for the duration of the follow-up period. Pregnant women will receive cash payments of 10,000 Tanzanian Shillings (~ $4.30) per antenatal care visit for up to four total visits. Mothers/caregivers will receive cash payments of 5000 Tanzanian Shillings (~ $2.15) per routine child health and growth monitoring clinic visit for up to one visit per month. Mothers/caregivers signed or provided thumbprints upon receipt of a cash transfer. The study coordinator will conduct random monthly spot-checks to ensure mothers/caregivers received CCTs as documented.

#### Control arm

Villages in the control arm will receive no intervention and have access to existing clinic-based services. No quality improvement activities will be undertaken by the study team.

### Data collection

**Fig. 2:**
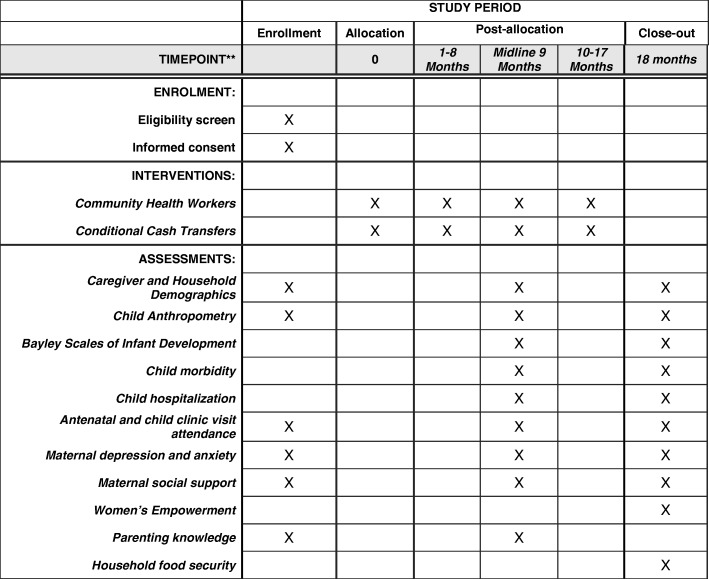
Schedule of enrollment, interventions and assessments (SPIRIT Figure)

The schedule of trial enrollment, interventions, and assessments is presented in Fig. 2. Trained fieldworkers will conduct home interviews with mothers/caregivers at baseline, midline (9 months of follow-up) and endline (18 months of follow-up). The fieldworkers will be blinded to the randomized intervention arm. At baseline, all enrolled mothers/caregivers will be administered a standardized questionnaire including demographic and socioeconomic data. Child length/height, weight, head circumference, and MUAC will be taken in triplicate at baseline, midline and endline at the home. Child weight will be measured to the nearest 100 g using a digital scale (Seca, Hamburg, Germany). Length of children < 24 months of age will be measured to the nearest 0.1 cm with a length board (Seca) and height of children ≥24 months will be measured to the nearest 0.1 cm with the use of a portable stadiometer (Seca). Anthropometric z-scores will be calculated using the 2006 WHO child growth standards [22]. At baseline, midline and endline RCH cards will be used to assess antenatal care and child health clinic visit attendance.

At baseline, midline and endline the Hopkins Symptom Checklist (HSCL-25) will be administered to assess symptoms of depression and anxiety [23]. Functional dimensions of social support will be assessed with the Duke University–University of North Carolina Functional Social Support Questionnaire [24]. The Caregiver Knowledge of Child Development Inventory (CKCDI) will be administered to mothers/caregivers at baseline and midline [25]. At endline, we will also assess women’s empowerment with components of the Women’s Empowerment in Agriculture Index (WEAI) [26] and household food security with the Household Hunger Scale [27]. Maternal caregiver recall of child morbidities in the last two weeks (diarrhea, fever, and other morbidities) and any child hospitalizations during the study period will be assessed at midline and endline. All of these modules have been used in previous research studies in Tanzania.

In order to assess child development at midline and end line, trained female research nurses will administer a Tanzania adapted and Swahili translated version of the Bayley Scales of Infant Development III (BSID-III) [18, 28]. The cognitive, expressive language, receptive language, fine motor, and gross motor subscales will be administered. We will select two BSID-III nurse assessors based on quantitative performance evaluations after a three-week BSID-III training, led by experts from Boston, USA. BSID-III composite scores for each development domain will be reported. The BSID-III assessors will be blinded to the participant’s allocated trial arm. In order to assess drift and ensure continued quality of implementation, the study coordinator will conduct weekly field-based supervision of BSID-III data collection at midline and endline.

### Statistical analyses

All primary analyses will be conducted based on the intention-to-treat (ITT) principle. Women who moved to neighboring villages will be analyzed in the village originally randomized. Despite randomization, it is possible that there may be some degree of imbalance of baseline prognostic factors between randomized trials arms due to the small number of clusters. As a result, we will re-assess intervention effects after adjusting for factors that are potentially imbalanced between randomized trial arms. All analyses will account for village clustering. We will use linear regression models to assess the intervention effect on individual-level BSID-III domain scores, HAZ, and other secondary continuous outcomes. We will use log-binomial models to assess treatment effects on binomial outcomes. Linear mixed effects models and generalized linear mixed models will be used to assess longitudinal continuous and binomial outcomes, respectively. We will also examine effect modification of any treatment effect by predefined baseline variables: maternal age, maternal education, maternal/caregiver parenting knowledge, parity, household wealth, marital status, paternal residence in household, maternal depression, maternal social support, child age, child sex, number of CHW visits received, and number of CCTs received. We will also present analyses collapsing the CHW and CHW + CCT arms.

### Ethics

The trial protocol was approved by the Ifakara Health Institute IRB (reference number 007–2017), the Tanzanian National Health Research Ethics Sub-Committee (NatHREC; reference no. NIMR/HQ/R.8a/Vol.IX/2538), and the Harvard T.H. Chan School of Public Health Institutional Review Board (reference no. IRB17–1001). Written informed consent will be sought from mothers and caregivers. The trial results will be disseminated locally in Tanzania and in international academic journals.

### Trial status

Trial enrollment started on 14 September 2017 and follow-up is ongoing as of 16 May 2019.

## Discussion

Sustainable Development Goal 4.2 calls for all countries to ensure that all girls and boys have access to quality early childhood development, care and pre-primary education by 2030 [29]. In order to address this call, we designed the current trial to examine the effect of a supply-side CHW interventions alone and in combination with a demand-side CCT intervention. These interventions were selected as Tanzania is currently in the process of implementing a national cadre of CHWs and CCTs have shown promise in the context of Latin America [14, 15]. The primary outcomes of the trial will be child development and child linear growth and we have included a range of maternal, child, and household secondary outcomes. This trial will contribute to the growing literature on integration of child health, nutrition, and responsive stimulation and social protection programs, particularly in the context of sub-Saharan Africa where the evidence is sparse.

There are a few limitations to the trial. As a proof-of-concept trial we will not have statistical power to detect small effect sizes on the primary or secondary outcomes of interest. Nevertheless, the comprehensive and integrated intervention package may provide large benefits. Second, we will only follow-up children for 18 months and as a result we cannot make conclusions about the long-term effects of CHWs and CCT interventions. Third, while the CCTs are designed to encourage clinic attendance, we did not provide quality improvement for clinic services and child growth monitoring. As a result, we may underestimate the potential effect of the CCTs.

This cluster randomized controlled trial will provide evidence whether CHW and CCT programs can be integrated to support child growth and development in rural Tanzania. The trial will also inform whether larger implementation evaluations should be pursued. The results of the trial are likely generalizable to similar rural contexts in sub-Saharan Africa that face a high burden of linear growth faltering and suboptimal child development.

## Data Availability

The datasets generated from the current study are available from the corresponding author.

## Abbreviations

BSID-III: Bayley Scales of Infant Development III
CCT: Conditional cash transfer
CHW: Community health worker
CKCDI: Caregiver Knowledge of Child Development Inventory
HAZ: Height-for-age z-score
HCAZ: Head circumference-for-age z-score
HDSS: Health and Demographic Surveillance
HSCL-25: Hopkins Symptom Checklist
IMCI: Integrated Management of Childhood Illness
LMIC: Low and middle-income countries
MUAC: Mid-upper arm circumference
SMD: Standardized mean difference
WAZ: Weight-for-age z-score
WEAI: Women’s Empowerment in Agriculture Index
WHZ: Weight-for-height z-score
